# Impact of an 8-week integrated neuromuscular training program on anthropometric and body composition profiles, jump-landing mechanics, and vertical jump height in male volleyball athletes—A non-randomized matched controlled study protocol

**DOI:** 10.3389/fspor.2026.1904088

**Published:** 2026-08-25

**Authors:** Vinosh Kumar Purushothaman, Siew Hong Foong, Albert Anand Udhaya Kumar, Watson Arulsingh, Arun Vijay Subbarayalu, Mariam Ameer, Praveen Kumar Kandakurti, Priyashini Batu Malai, Rajkumar Krishnan Vasanthi

**Affiliations:** 1 Faculty of Health & Life Sciences, INTI International University, Nilai, Negeri Sembilan, Malaysia; 2 Out Patient Physical Therapy Department, Cleveland Clinic, Abu Dhabi, United Arab Emirates; 3 Department of Physiotherapy, College of Health Sciences, Gulf Medical University, Ajman, United Arab Emirates; 4 College of Education, Administrative and Technical Sciences, Arabian Gulf University, Manama, Bahrain; 5 Department of Biomechanics, Faculty of Physical Therapy, Cairo University, Giza, Egypt

**Keywords:** anthropometric characteristics, body composition metrics, health risk, jump landing, lower limb power, movement mechanics, volleyball athlete

## Abstract

**Introduction:**

Efficient neuromuscular coordination, appropriate landing and lower limb strength is a key factor in volleyball performance. Non-ideal body composition and poor landing may lead to injury risk and poorly affect the performance. This study aims to investigate the effect of 8-weeks integrated neuromuscular training program on anthropometric characteristics, body composition, jump-landing biomechanics, and vertical jump height among adult male volleyball athletes.

**Methods:**

This non-randomized matched controlled study will utilize 28 male volleyball players aged between 18 and 25 years. Following baseline assessment, participants will be allocated to experimental group (INMT along with volleyball training) and control group (volleyball training along with a structured resistance training program) using non-randomized allocation procedure and a set of predefined criteria. The intervention will last 8 weeks, with three sessions per week. Primary outcome parameters such as body composition, anthropometric profiles, landing kinematics will be assessed using bioelectrical impedance analysis and landing error scoring system. The secondary outcome, vertical jump height, will be analyzed using vertec jump tester respectively. Group × time interaction test will be computed using ANCOVA, adjusted for baseline/linear mixed-effects model. Secondary outcome *p*-values will be adjusted for multiplicity, using the Holm-Bonferroni method.

**Discussion:**

To address the gap in the literature, this study will examine whether INMT improves anthropometric and body composition profiles, jump-landing mechanics, and vertical jump height in male volleyball athletes. By testing at 8 weeks, the study may provide data on the velocity of neuromuscular adaptations, validating whether significant changes occur within this specific periods. In addition, the findings may contribute to the specific profiling of male volleyball athletes by establishing how INMT affects both body composition and biomechanical metrics (jump height, Landing Mechanics) specifically in this population.

**Clinical Trial Registration:**

ClinicalTrials.gov, identifier, NCT07485348.

## Introduction

The performance in volleyball is characterized by dynamic, fast paced game with high biomechanical demands, frequent jumping multidirectional movements coupled with precise neuromuscular coordination (1). Both offensive and defensive phases entail intricate movement patterns, with the spike recognized as one of the most critical offensive skills. During a spike, athletes strive to attain optimal vertical elevation to establish a superior point of attack and enhance unpredictability. Improved vertical leap ability facilitates a more acute and powerful trajectory, maximizing scoring potential. Attacking jump is a crucial factor for enhancing performance in volleyball and the height attained during the offensive phase also contribute to the outcomes of volleyball game in both male and female athlete (2).

Anthropometric profiles are significantly related to athletic performance and are employed to examine athletes in various sports with physical growth recognized as essential for attaining outstanding athletic achievements (3). Attributes including increased height, reduced body fat percentage, and lean muscular mass substantially enhance jumping performance and agility (4). These attributes may affect joint kinematics and force production during explosive movements, improving biomechanical efficiency and mitigating the risk of overuse injuries. Comprehending the interplay between anthropometric variables, neuromuscular control, and jump-landing mechanics is essential for developing effective integrated training intervention protocol that incorporate strength, and proper landing training may improve performance and injury resilience in volleyball athletes (5).

Essential anthropometric measurements encompass stature, body mass, body mass index (BMI), limb length, and body circumference parameters, which are frequently utilized to assess body composition, adiposity, and skinfold thickness. In athletic populations, these metrics are utilized to assess body composition, monitor health status, and detect potential medical issues, including energy deficit and eating disorders (6) In addition to unchangeable characteristics like height and bone structure, the ratio of fat-free mass to fat mass significantly influences performance abilities. Athletes possessing reduced fat mass and increased lean body mass generally exhibit enhanced strength-to-mass ratios, power output, and aerobic endurance (7). Recent evidence demonstrated that agility test was significantly higher in athletes with higher muscle mass. In the context of endurance, athletes with greater lean mass presented with high level of cardiovascular fitness and recovery capacity. On the other hand, higher body fat percentage is correlated with slower sprint and agility task (8). Therefore, optimal body composition is vital to enhance movement efficiency and reaction speed during competition. Volleyball players are especially prone to musculoskeletal injuries, with the knee, ankle, and shoulder recognized as the most commonly affected areas (9). A study of the Brazilian men's national volleyball team from 2018 to 2021 recorded 28 injuries affecting 12 out of 41 players. The knee had the largest share of injuries at 11/1,000 players, succeeded by the ankle at 69/1,000 athletes (10).

These data highlight the considerable injury burden linked to the repetitive movements of jumping, landing, and spiking that characterize elite volleyball performance. Volleyball players face a significant risk of lower-limb injuries due to the repetitive nature of jumping and landing in the sport (11). During landing, it is essential to efficiently disperse the kinetic energy produced during take-off to reduce stress on the joints and soft tissues. Enhancing landing biomechanics by increasing knee flexion and regulating motion diminishes kinetic stress and improves energy absorption.

Neuromuscular and plyometric training regimens that include strength, balance, and stability exercises have demonstrated efficacy in enhancing lower-limb control and reducing injury risks among volleyball athletes (12). Neuromuscular Training (NMT) is a systematic program designed to enhance performance and mitigate injury risk by incorporating fundamental functional movement skills, that concentrate exclusively on neuromuscular control and coordination (13, 14). In contrast, integrated neuromuscular training (INMT) primarily focuses on an advanced training approach that incorporates sports specific drills and motor task to enhance athletic performance (15). Furthermore, data from systematic review and meta-analysis indicate that NMT enhances lower-limb alignment during landing task and may mitigate intrinsic risk factor for injury in volleyball athletes. These data collectively endorse that incorporating INMT protocol may improve power, injury resilience and landing mechanics in volleyball players (16).

Although numerous studies have evaluated the effectiveness of neuromuscular or plyometric training, most investigations have focused on isolated performance outcomes**,** such as vertical jump height, balance, or landing biomechanics, rather than examining multiple interrelated adaptations simultaneously (17, 18). Furthermore, much of the available evidence has been derived from basketball, soccer, and mixed-sport populations or from elite adolescent athletes, thereby limiting the generalizability of findings to adult male volleyball athletes (18, 19)**.** Previous volleyball-specific studies have primarily emphasized improvements in jump performance or injury prevention, with comparatively limited attention given to whether neuromuscular adaptations are accompanied by concurrent changes in anthropometric characteristics, body composition, and movement quality following a structured INMT program (20).

Evaluating these variables collectively is clinically and practically important because they represent complementary domains influencing volleyball performance and injury risk. Anthropometric characteristics and body composition affect force production, movement efficiency, and relative power output, whereas landing biomechanics reflect neuromuscular control and dynamic knee stability during high-impact tasks. Vertical jump height represents explosive lower-limb power, a critical determinant of volleyball performance. Since these factors interact biomechanically and physiologically, examining them within a single intervention provides a more comprehensive understanding of the adaptations induced by INMT than evaluating each outcome independently (21, 22).

In addition, INMT produces significant neuromuscular and physiological adaptations in volleyball players, such as increased flexibility, power, stability, motor competence and physical performance (23, 24). As such, this INMT incorporates various elements like motor skill development, core stability, balance and strength, all of which have a positive impact on athletic performance. Therefore, this study will aim to investigate the effects of an eight-week Integrated Neuromuscular training program on anthropometric characteristics, body composition, jump-landing biomechanics, and vertical jump height among adult male volleyball athletes. We hypothesized that structured INMT will produce significant improvements across these interrelated performance and biomechanical outcomes. The findings are expected to assist physiotherapists, strength and conditioning specialists, and volleyball coaches in designing evidence-based, sport-specific injury prevention and performance enhancement programs.

## Methodology

### Study design & ethical approval

This study will utilize a non-randomized matched controlled study approach to assess the impact of an eight-week integrated neuromuscular training (INMT) program on the anthropometric profile, jump-landing mechanics, and vertical jump height of young male volleyball athletes. Given the non-randomized matched controlled study, causal interpretation will be limited. Matching and baseline adjustment procedures will be used to reduce, but not eliminate, potential selection bias. However, certain methodological measures will be implemented to augment internal validity and to minimize bias. The participants baseline characteristics will be assessed to establish group comparability and minimize the confounding emerges from assessed variables. In addition, to reduce bias in the observation and measurement, the assessor who measured the outcome parameters will be blinded during the grouping and data collection phase (25). Participants will be divided into two groups: an Experimental Group (EG) that will receive INMT thrice a week for eight weeks, and a Control Group (CG) that maintain their standard volleyball training regimen. Despite the absence of random allocation, both groups will be comparable in baseline parameters such as age, height, weight, and body mass index (BMI). Purposive sampling will be utilized to enlist experienced volleyball players who satisfied certain inclusion criteria, guaranteeing adequate baseline proficiency and conditioning for the intervention.

This study will adopt a matched controlled allocation approach to facilitate the matching between the groups. When random allocation is not feasible, non-random grouping with a matching baseline characteristic can be employed as it is well established method to compare groups (26). Participants will be matched based on the key baseline data such as such as age, gender, training periods, training duration and frequency, playing position, competitive level to maintain the uniformity as these factors will be perceived as potential confounders that may affect the result and balancing performance. Matching will be carried out prior to group allocation by pairing participants with identical baseline characteristics to guarantee the balance between the groups. Group allocation will be based on club accessibility and training logistics rather than random selection. Subsequently, after allocation, baseline homogeneity will be assessed using ANCOVA or Friedman test for continuous variable based on distribution of data and chi-square test for categorical variables. The standardized mean difference will be computed to examine the extent of any remaining baseline imbalance. If significant baseline difference exists after matching the appropriate variable will be included as covariates in the primary analyses to minimize the residual confounding. To mitigate expectation effects, the outcome assessor will be blinded to the group allocation, and the statistical analyses will be employed using data coding procedure to explicit the exposure of group allocation.

The study will be conducted at private volleyball clubs located in Nilai and Subang, Malaysia. Ethical approval has been obtained from the University Research and Ethics Panel with ref. no. INTI-IU/FHLS-RC/BPHTI/1NY12025/007 and adhered to the declaration of Helsinki. This non-randomized matched controlled study has been registered with ClinicalTrials.gov (NCT07485348). The exercise intervention will be delivered by a sports physiotherapist who had over 10 years of experience in the relevant field. All participants will be briefed about the study's objectives and procedures. The participation of the subjects in this study will be voluntary, and each participant would be expected to sign the informed consent form before participation. Subjects will be allowed to discontinue from the study if they experience any form of musculoskeletal injury or any adverse effects observed during the delivery of the intervention. Any form of harm during the session will be documented through observation and self-reporting measures.

### Sample size and participants

The target population will be comprised of male volleyball players aged 18 to 25 years, with a minimum of one year of continuous playing experience at university level, playing position setter, hitter, blocker, a training frequency of at least twice per week with minimum weekly training volume of 3 h, previous lower limb injury status will be collected. Individuals who had sustained a recent lower-limb injury or undergone surgery within the past six months, individuals with neurological or vestibular diseases, or participants involved in other structured training programs will be excluded. This selection guaranteed a uniform athletic background among participants. LESS, anthropometric measures, and body composition will be considered as primary outcomes, and vertical jump height will remain as a secondary outcome. Sample size has been calculated using G*Power (version 3.1.9.4) based on effect sizes obtained from research conducted by Pfile et al., which demonstrated a significant change in LESS after 6 weeks of NMT (F^2^, ^10^ = 6.29, *p* = 0.009) providing a partial eta square (*η*^2^p) of 0.557 and cohen's f of 1.12 (27). However, this study utilized single-group repeated measures instead of a controlled trial, and a more cautious interaction effect size (f = 0.25) was computed using ANCOVA, adjusted for baseline/linear mixed-effects model. Secondary outcome *p*-values will be adjusted for multiplicity, using the Holm-Bonferroni method with a significance level of *α* = 0.05 and statistical power of 0.8, which necessitated a minimum of 28 participants (14 per group). The total recruiting target will be established at 31 athletes to account for a probable 10% dropout. This sample size calculation has been performed considering the primary outcome only; results regarding secondary outcomes should be considered exploratory.

## Outcome parameters

### Anthropometric profile and body composition

The anthropometric profile will be assessed utilizing a Bodystat 1500MD bioelectrical impedance analysis (BIA) instrument, which quantifies body composition metrics including body fat percentage, lean mass percentage, total body water (TBW%), BMI, and waist–hip ratio (WHR). Bioelectrical impedance analysis (BIA) is a non-invasive and dependable technique for evaluating body composition, exhibiting moderate to high reliability (ICC = 0.67–0.99) and validity in estimating fat-free mass within athletic populations (28). Nevertheless, given that hydration, ethnicity, and sex can affect impedance measurements, all participants will be directed to sustain normal hydration, refrain from vigorous activity for a minimum of 48 h and follow their customary diet before testing. Participants will complete a standardized pre-assessment questionnaire before each assessment to ensure that they will be well-hydrated, will avoid intensive exercise for 48 h prior to the assessment, and will maintain their normal food intake. Compliance will be verbally verified by the investigator before testing. Weight and height will be assessed with a calibrated stadiometer, and BMI will be computed appropriately.

### Landing mechanics

Jump-landing mechanics will be assessed using the Landing Error Scoring System (LESS). LESS consists of 17 landing qualities that will be observed during both the initial contact and the entire landing phase. This system has shown excellent intrarater (ICC = 0.82–0.99) and interrater reliability (ICC = 0.83–0.92) as well as good concurrent validity when compared to three-dimensional motion analysis (29). Participants will execute a standardized jump-landing manoeuvre by jumping forward from a platform 30 cm in height to a distance equivalent to 50% of their body height from the platform's front edge. Participants will be instructed to jump directly forward from the 30 cm-high platform, reducing vertical displacement. Upon landing, the participants will be directed to promptly retract and do a rebound jump to achieve optimal vertical elevation. Participants then will be directed to execute the initial jump off the platform and the subsequent rebound jump in a seamless manner. Participants will therefore perform three trials of a standardized jump-landing task during the testing phase; to minimize the fatigue a period of 30 s rest period will be provided between each jump. To familiarize themselves with the technique, participants will be allowed to perform 2 practice trial jumps before the actual testing procedure. Joint motions will be recorded using two standard high-definition Nikon D7100 DSLR camera with a resolution of 1920 × 1,080 at 30 frames per second. Prior to data acquisition, the setup will be calibrated with standardized reference grid to ensure the camera alignment and filed of view. Cameras will be positioned three meters in front (frontal plane) and to the right (sagittal plane) from the landing area, with camera height aligned 1.2 meter from the floor. The videos will be assessed later by the LESS trained assessor to identify the 17 jump landing motion errors. The scoring assessor will be provided formal training session on scoring method using LESS scoring manual and instructions. Prior to the commencement of the study, the assessor will practice the scoring system through a trial recording. The recordings will be processed with Kinovea motion analysis software, enabling meticulous frame-by-frame evaluation of joint angles and landing style by the same assessor.

### Jump height

Vertical jump height will be determined using the Vertec jump tester, a reliable field-based device widely used in sports performance assessments. The Vertec has demonstrated strong correlation with force-platform and flight-time methods (r = 0.86–0.93) and high test–retest reliability (ICC = 0.73–0.99). Participants’ standing reach height will be measured first, followed by three maximal countermovement jumps separated by two-minute rest intervals. The highest vane displaced will be recorded and used for analysis. Although the Vertec may produce slightly higher readings than force-platform measures, it remains a consistent and valid tool when standardized protocols are followed (30).

Data collection will be conducted at two time points: pre-test (baseline) and post-test (after eight weeks). Participants will complete the demographic questionnaire covering age, training history, injury background, and weekly training frequency. All assessments will be conducted under identical conditions by a trained assessor who will be blinded about group allocation to maintain measurement consistency. Each test will be interspersed with a standardized two-minute rest period to mitigate tiredness. Data confidentiality will be rigorously maintained during the investigation. The order of testing will follow the sequence for all participants: Demographic questionnaire, anthropometric and body composition measures, the jump-landing assessment, and then the vertical jump height testing. Unique participant identification numbers will be used to code the data for confidentiality and for effective data management. Measurement procedures will be standardized, with calibrated instrumentation and the same environmental setting. Further, blinding the assessor to the group allocation, and uniform verbal instructions, will be used to reduce measurement bias. Recorded data will also be randomly checked to ensure accuracy and completeness.

## Intervention protocol

The intervention for both groups will have a matched warm up and cool down phase of 10 min and 5 min respectively along with 45 min of structured exercise protocol, a 1-minute rest interval will be provided between each set for both groups. The intervention regimen for the experimental group comprised an 8-week structured neuromuscular training program conducted thrice a week, in conjunction with regular volleyball practice. Each 60-minute session will commence with a 10-minute dynamic warm-up that incorporated mobility and stretching exercises, including lunges with trunk rotation, leg swings, and jogging. Borg scale of rate of perceived exertion (RPE) will be used to prescribe the exercise intensity for both groups. The exertion level will be maintained between 13 and 16 (somewhat hard to hard). RPE will be recorded after each training session and utilized to guide progression when appropriate. Training load during each session will be closely monitored through RPE method (session × perceived exertion score). The primary training phase will include 45 min of incremental neuromuscular activities aimed at improving proprioception, balance, and lower-limb control. In the initial four weeks (Foundation phase), participants will be instructed to execute bodyweight squats, bilateral stance on BOSU ball, unilateral stance with contralateral leg swing, Jump and freeze, Lateral bounds, Single Leg Romanian Deadlift. Progression of the training load will be implemented every 4 weeks by modifying exercise complexity, balance challenge, repetitions and external resistances. Each progression will be allowed only when participants performed correct technique without any compensatory movement patterns during two consecutive sessions. The training intensity and complexity will be incrementally elevated from weeks five to eight (Advanced neuromotor phase) through the incorporation of barrier Jump front cone runs, Single leg hops (lateral + forward) and Zig-zag cone drills, Single Leg Romanian Deadlift. A one-minute rest interval will be provided between sets, and each session will be concluded with a 5-minute cool-down comprising light jogging and static stretching [EG training Protocol is specified in Table 1]. In contrast, the CG will continue their routine volleyball training supplemented with a structured resistance-training program over the same period. Approximately 60 min per session [CG training Protocol is specified in Table 2]. This design will ensure that any observed differences between groups could be attributed to the neuromuscular component rather than general training volume or duration. The training protocol for EG will be adopted and modified from the previous studies (31–33). The training regimen for CG will be derived from validated earlier protocols in volleyball specific conditioning studies (34, 35). Participants will be instructed not to engage in any other structured interventions that are designed to improve body composition and movement patterns during the intervention session. To maintain the fidelity, all the sessions will adhere to a standard training manual, consistent exercise sequence, progression timeline. All training sessions will be conducted at the indoor volleyball training facility centre equipped with a suitable training surface, and the intervention will be closely supervised by the qualified sports physiotherapist to ensure proper exercise execution, deliver feedback and minimize injury risk. A 85% regular attendance rate will be mandatory to comply with the intervention protocol. Adverse events such as musculoskeletal strain, sprains or any other form of sports related incidents will be recorded and reported to ethical committee by the principal investigator throughout the study. In addition, upon completion of the intervention protocol, all outcome parameters will be measured immediately on the same day using a randomized test sequence for all groups, with the same investigator being assigned to collect data to avoid bias of the assessor between the pre- and post-intervention period in the same environment.

**Table 1:** Protocol for experimental group.

Week	Phase	Exercise	Sets	Repetitions/ Duration	Rest Interval	Intensity	Progression Criteria	Supervision	Safety Notes
Weeks 1–8 (3 sessions/week)	Warm-up (all sessions)	Dynamic stretching	1	10 min	N/A	Low	None (fixed component of every session)	Physiotherapist-supervised	Perform within pain-free range; ensure adequate warm-up before progressing to conditioning exercises
Weeks 1–8 (3 sessions/week)	Cool-down (all sessions)	Light jogging + static stretching	1	5 min	N/A	Low	None (fixed component of every session)	Physiotherapist-supervised	Hold stretches for 20s within pain-free range; monitor for post-exercise dizziness or discomfort
**Neuromotor Foundation Phase — Weeks 1–4 (Low-to-moderate intensity)**									
1–4	Session 1: Balance & Plyometrics	Bilateral stance on BOSU ball; unilateral stance with contralateral leg swing (40° flexion–extension); heel raise and balance on one knee on BOSU ball; balancing while catching a tennis ball	3	30 s/leg	1 min between sets	Low–moderate	Progress to Weeks 5–8 protocol once balance is maintained without loss of postural control or compensation	Direct supervision by physiotherapist	Non-slip mat under BOSU ball; physiotherapist spots throughout to prevent falls
		Jump and freeze	3	10 reps	1 min between sets	Low–moderate	Increase landing control demand before advancing to Weeks 5–8		Land softly with knees soft; therapist checks landing mechanics
		Lateral bounds	3	10 reps	1 min between sets	Low–moderate	Increase distance/speed before advancing to Weeks 5–8		Clear surrounding area; supportive footwear required
		Squat jump	3	10 reps	1 min between sets	Low–moderate	Progress jump height/complexity before advancing to Weeks 5–8		Cue soft landing; therapist monitors knee alignment
		Ankle hops	3	10 reps	1 min between sets	Low–moderate	Progress repetitions/speed before advancing to Weeks 5–8		Perform on non-slip surface; stop if ankle discomfort occurs
	Session 2: Agility & Strength Training	T-drill	3	4 reps	1 min between sets	Low–moderate	Advance to Weeks 5–8 multidirectional drills once technique is consistent		Clear drill area of obstacles; appropriate footwear
		4-corner sprint drill	3	4 reps	1 min between sets	Low–moderate	Advance to Weeks 5–8 multidirectional drills once technique is consistent		Ensure adequate spacing between participants
		Ladder feet drill	3	4 reps	1 min between sets	Low–moderate	Advance tempo before progressing to Weeks 5–8		Check ladder is secured flat on floor
		Lunges (front & back)	3	10 reps	1 min between sets	Low–moderate	Progress load/range before advancing to Weeks 5–8		Monitor knee tracking over toes
		Bilateral calf raises on step	3	10 reps	1 min between sets	Low–moderate	Progress to Weeks 5–8 loading		Hold support rail if balance is unsteady
		Single-leg Romanian deadlift	3	10 reps/leg	1 min between sets	Low–moderate	Progress to Weeks 5–8 loading once form is consistent		Maintain neutral spine; therapist corrects hip hinge pattern
**Advanced Power and Neuromotor Phase — Weeks 5–8 (Moderate-to-high intensity)**									
5–8	Session 1: Balance & Plyometrics	Bilateral stance on BOSU ball; unilateral stance with contralateral leg swing (40° flexion–extension); heel raise and balance on one knee on BOSU ball — each exercise performed while holding a 1 kg medicine ball	3	30 s/leg	1 min between sets	Moderate–high	A 1 kg Bulgarian bag/medicine ball was added for the balance exercises; repetitions and complexity of plyometric components were increased relative to Weeks 1–4	Direct supervision by physiotherapist	Non-slip mat under BOSU ball; therapist spots throughout to prevent falls
		Barrier jump (front/back, side to side)	3	12 reps	1 min between sets	Moderate–high	Increased repetitions vs. Weeks 1–4 equivalent drills		Barrier height matched to participant capability; spotter present
		Single-leg hops (lateral + forward)	3	12 reps	1 min between sets	Moderate–high	Increased repetitions vs. Weeks 1–4 equivalent drills		Ensure adequate space; land softly with knee flexion
		Single-leg box jump	3	5 reps/leg	1 min between sets	Moderate–high	New exercise introduced in this phase; box height progressed only when landing control is consistent		Box height appropriate to participant capability; spotter present; soft landing surface
		Scissor jumps	3	12 reps	1 min between sets	Moderate–high	New exercise introduced in this phase		Cue controlled landing; stop if knee/hip pain occurs
		Tuck jump with landing control	3	12 reps	1 min between sets	Moderate–high	New exercise introduced in this phase; emphasis on soft, controlled landing		Therapist checks landing mechanics each set; stop if form deteriorates
	Session 2: Agility & Strength Training	Zig-zag cone drill	2	10 reps	1 min between sets	Moderate–high	Multidirectional change-of-direction demand prioritized relative to Weeks 1–4 drills		Clear cone spacing; appropriate footwear
		Multi-direction random cone drill	2	10 reps	1 min between sets	Moderate–high	Multidirectional change-of-direction demand prioritized relative to Weeks 1–4 drills		Ensure adequate space for unpredictable movement directions
		Ladder tempo drills	2	10 reps	1 min between sets	Moderate–high	Tempo increased relative to Weeks 1–4 ladder drill		Check ladder is secured flat on floor
		Bilateral calf raises on step	3	10 reps	1 min between sets	Moderate–high	Strengthening performed with a Bulgarian bag weighing 5% of body mass		Hold support rail if balance is unsteady
		Single-leg Romanian deadlift	3	10 reps/leg	1 min between sets	Moderate–high	Strengthening performed with a Bulgarian bag weighing 5% of body mass		Maintain neutral spine; therapist corrects hip hinge pattern

**Table 2:** Protocol for control group.

Week	Phase	Exercise	Sets	Repetitions/ Duration	Rest Interval	Intensity	Progression Criteria	Supervision	Safety Notes
Weeks 1–8 (3 sessions/week)	Warm-up (all sessions)	Dynamic stretching	1	10 min	N/A	Low	None (fixed component of every session)	Direct supervision by physiotherapist	Perform within pain-free range
Weeks 1–8 (3 sessions/week)	Cool-down (all sessions)	Light jogging + static stretching	1	5 min	N/A	Low	None (fixed component of every session)		Hold stretches for 20s within pain-free range
**Strength Training Phase 1 — Weeks 1–4 (Moderate intensity)**									
1–4	Resistance Training	Barbell hip thrust	3	10 reps	1 min between sets	Moderate	Load progressed to Weeks 5–8 exercise variation once technique is consistent	Direct supervision by physiotherapist	Spotter present; verify bar/pad positioning before loading
		Leg press	3	10 reps	1 min between sets	Moderate	Load progressed to Weeks 5–8 exercise variation once technique is consistent		Confirm seat/carriage setting; avoid locking knees at end range
		Romanian deadlift	3	8 reps	1 min between sets	Moderate	Progressed to single-leg variation in Weeks 5–8		Maintain neutral spine; therapist corrects hip hinge pattern
		Barbell back squat	3	8 reps	1 min between sets	Moderate	Progressed to front squat/half-squat variations in Weeks 5–8		Spotter present; monitor knee tracking and lumbar position
		Standing bilateral heel raise	3	10 reps	1 min between sets	Moderate	Load progressed in Weeks 5–8		Hold support rail if balance is unsteady
**Strength Training Phase 2 — Weeks 5–8 (Moderate-to-high intensity)**									
5–8	Resistance Training	Walking dumbbell lunges	3	12 reps/leg	1 min between sets	Moderate–high	Increased load/volume relative to Weeks 1–4 lunge pattern	Direct supervision by physiotherapist	Ensure clear walking path; monitor knee tracking
		Deadlift	4	8 reps	1 min between sets	Moderate–high	Increased sets and load relative to Weeks 1–4 Romanian deadlift		Spotter present; maintain neutral spine throughout lift
		Single-leg glute bridging	3	12 reps/leg	1 min between sets	Moderate–high	New unilateral variation introduced in this phase		Maintain pelvic alignment; stop if lumbar discomfort occurs
		Front squat	3	12 reps	1 min between sets	Moderate–high	New squat variation introduced in this phase		Spotter present; monitor bar rack position and torso angle
		Barbell half squat	3	12 reps	1 min between sets	Moderate–high	New squat variation introduced in this phase		Spotter present; control depth to avoid excessive load on knees
		Single-leg Romanian deadlift	3	12 reps	1 min between sets	Moderate–high	Progressed from bilateral Romanian deadlift in Weeks 1–4		Maintain neutral spine; therapist corrects hip hinge pattern

### Data analysis

Data will be analysed using SPSS version 29.0 (IBM Corp., Armonk, NY, USA). Descriptive statistics will be presented as means and standard deviations for all dependent variables. Multiple imputation analysis will be implemented to evaluate the robustness of the findings. The primary analysis will be a group × time interaction test performed using ANCOVA, adjusted for baseline/linear mixed-effects model (LME), and will report effect sizes and 95% CIs for all outcomes. Secondary outcome *p*-values will be adjusted for multiplicity, using the Holm-Bonferroni method. The primary analysis population will be the intention-to-treat population, with multiple imputation to handle missing follow-up data, and a per-protocol analysis (at least 85% session attendance) will be conducted as a sensitivity analysis. Treatment group, assessment time points, baseline levels of all outcome measures, demographic variables (age, body mass index), and missingness and/or outcome measure related variables will be part of the imputation model. Sensitivity analyses will be conducted to assess the robustness of the findings: (1) multiple imputation ITT analysis, (2) complete case analysis, and (3) per protocol analysis (attending ≥85% of intervention sessions).

The principal researcher and the team will frequently monitor the subject's safety, adherence to protocol, data collection. No interim analysis will be implemented as the study will be investigated throughout the intervention period. Furthermore, data monitoring committee will not be formed for this study, as the study involves shorter duration, non-invasive outcome measurements and relatively low-risk intervention. However, safety of the participants will be closely monitored by the principal investigator and the team throughout the study period. Any adverse events, injuries during the intervention will be documented, managed and reported to ethics committee if required.

## Discussion

The current non-randomized matched controlled study protocol is to create an efficient eight-week integrated neuromuscular training program that tends to enhance volleyball players’ anthropometric, body composition profiles, landing mechanics and vertical jump height. Although earlier studies have examined the association between anthropometric profiles, landing mechanics, jump height and performance metrics in volleyball, majority have investigated these variables in isolation rather than as interconnected elements of athletic performance (36). While previous study analyzed jump-landing mechanics under various conditions such as ball position, joint loading and screening reliability, associated with volleyball spike did not test the influence of anthropometric variable on this landing mechanics, nor guided to the most suitable intervention to control these covariates (37, 38). Moreover, there is a paucity in research that concurrently addresses changes in anthropometric variables, body composition, landing kinematics and performance after systematic INMT regimen. Therefore, the present study protocol aims to address this gap by evaluating the effects of an 8-week integrated neuromuscular training program on anthropometric, body composition variables, vertical jump height and jump-landing mechanics in male volleyball athletes.

Moreover, this study planned to utilize non-randomized matched controlled study design because of practical constraints encountered in team sports settings in terms of preserving team cohesion and existing training schedules (39). Participants will be matched based on key baseline characteristics, including age, sex, playing experience, and baseline performance measures, before group allocation. This matching procedure is intended to ensure that the study groups are comparable at baseline and to minimize potential selection bias resulting from differences in participant characteristics. Consequently, any observed differences in outcomes are more likely to be attributable to the intervention rather than pre-existing group differences. Furthermore, Lam et al. recommended that the intervention can be implemented within the participants’ routine training environment to enhance the ecological validity of the study and to increase the applicability of the findings to real-world sports practice (40).

Furthermore, this non-randomized matched controlled study approach is acceptable to applied sports science research, where complete randomization may be a challenging task. By addressing gaps in the existing literature, this study may contribute to the evidence base for INMT over anthropometric variables and skill tests in volleyball which may draw helpful information to coaching practices, to make injury prevention strategies, and performance enhancement programs. Additionally, an 8-week intervention duration selected for our study is based on previous neuromuscular training studies which have yielded a significant improvement in jump performance and landing mechanics within this timeframe (36, 41). The first four weeks of integrated neuromuscular training program will concentrate on plyometric and balance training, which tends to improve strength, speed, balance, and explosive force—all of which are characteristics of volleyball playing and crucial for sportive performance. This is because speed and balance are the most essential basic motoric features in volleyball (42). The second four weeks of the program will focus on a progressive balance and plyometrics to enhance performance as well as gradual increment in agility and strength training to reduce the risk of injury and enhance a player's ability to swiftly shift direction when necessary for gaming (43, 44). The integration of plyometric exercises (e.g., drop jumps, hops) will increase tendon stiffness and improve the stretch-shortening cycle, allowing athletes to store and release more elastic energy for higher jumps. The training, especially if it includes single-leg components, can address unilateral imbalances (differences in strength or landing between legs), thereby reducing injury risk (16).

Additionally, a high-quality study reported a significance of this INMT on vertical jump height over controls and indicated correlation between maturity and the performance skill of jumping in youth volleyball players (32). All training sessions will be monitored for attendance, adherence which in turn be supervised by qualified therapist and coaches. Adverse events will be monitored week wise and documented and reported as per the institutional protocols. All data will be deidentified and stored securely in accordance with institutional data protection policies and guidelines. To the best of our knowledge, few studies have examined the effects of an INMT program on physical performance, our study will focus exclusively on anthropometric profiles, body composition, jump-landing mechanics, and vertical jump height among volleyball players.

However, evidence regarding the effects of integrative neuromuscular training (INMT) on these kinetic chain outcomes remain limited. The current study protocol is expected to fill an important gap in the literature by adding a structured, replicable intervention targeting multiple key attributes and performance lead to injury-related outcomes.

### Limitations

Although this study may have limitations such as a small sample size and a compromised effect size, it is likely to provide feasibility data and serve as a platform for future prospective randomized controlled trials to further test the hypothesis. Also, the study involves only male players, which may potentially limit the significance of results to other groups of athletes. Further, evaluation of Body composition using BIA can be less accurate than standard methods like DXA. Additionally, LESS could be less accurate for a detailed biomechanical metrics than a 3D motion analysis.

### Practical implications

The findings of this study protocol may provide coaches, strength trainers and conditioning specialists, sports physiotherapists with a structured, evidence-based and replicable training strategy that can be incorporated into a normal training session to improve the biomechanics of volleyball game and prevent the injury rate. The protocol outlined in this study could be used as a practical guideline for in-season and pre-season conditioning and could be modified for other athletes that jump and land often. Finally, the results of this study protocol is expected to provide practical evidence for the effectiveness of 8-INMT to enhance the body composition, jump-landing mechanics and vertical jump performance in male volleyball players.

## Conclusion

This protocol will provide preliminary evidence whether incorporation of an 8-week integrated neuromuscular training program can improve landing mechanics, vertical jump performance, anthropometric and body composition-related outcomes in male volleyball athletes in addition to regular training program.

## Data Availability

The data will be available from the corresponding author on reasonable request. The output of the results will be declared in trial registry portal after completion of the project.
